# Johannes Schmiedt (1623–1690)

**DOI:** 10.1007/s00415-021-10602-w

**Published:** 2021-05-13

**Authors:** Katarzyna Pekacka-Falkowska, Bartlomiej Siek

**Affiliations:** 1 Poznan University of Medical Sciences, Przybyszewskiego 37a, 60365 Poznan, Poland; 2 Medical University of Gdansk, Marii Sklodowskiej-Curie 3a, 80210 Gdansk, Poland

**Fig. 1:**
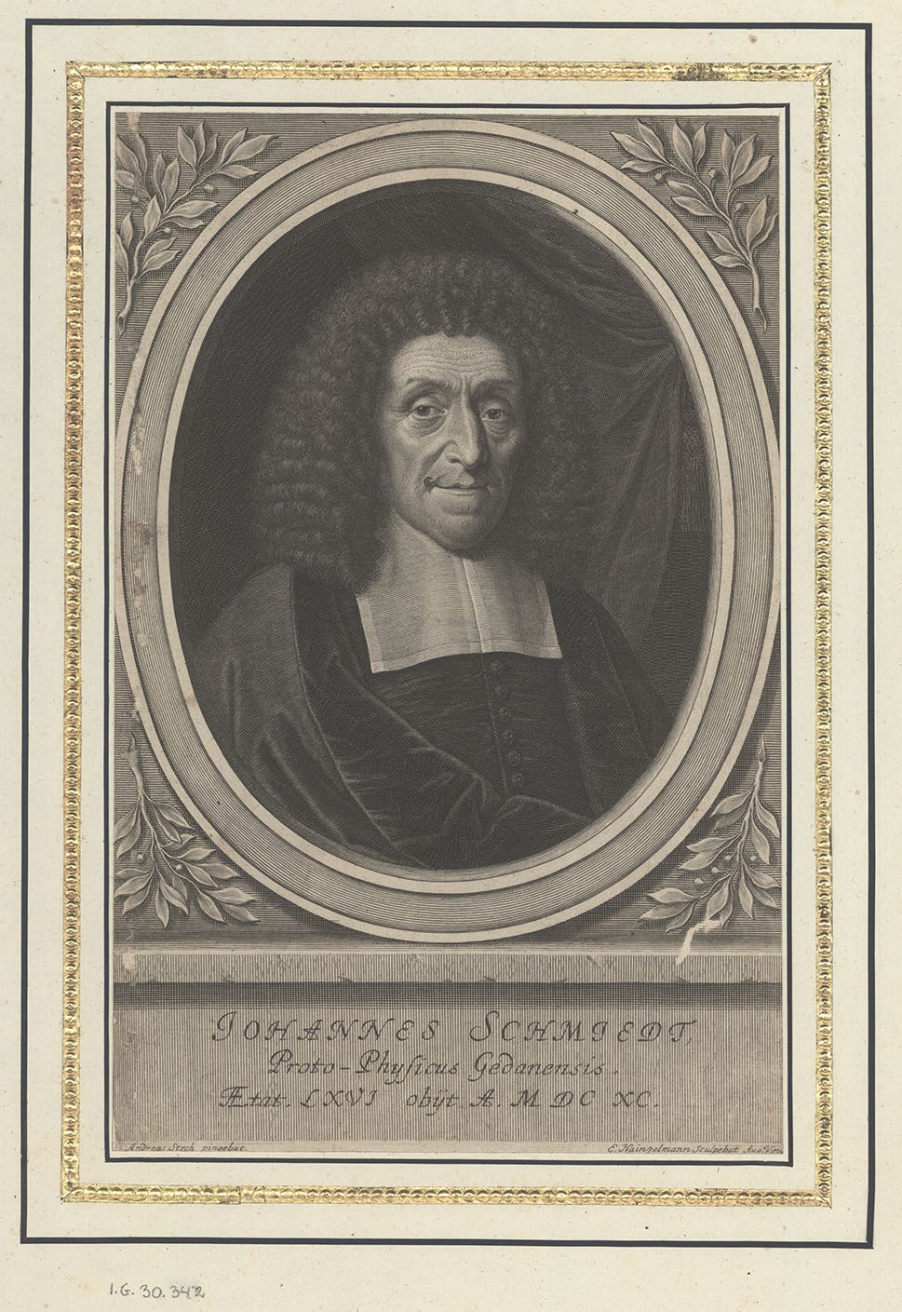
Johannes Schmiedt. Courtesy of The National Digital Library Polona (Poland)

Johannes Schmiedt (also known as Fabricius) (Fig. 1) was born on December 1st, 1623, in Danzig (present-day Gdańsk). His father, Daniel, was a municipal physician and a well-respected medical practitioner, whereas his mother, Catharina née Schevecke, was the daughter of a city councillor. He had six siblings.

In his early years, Schmiedt received private tuition in Latin, German and Polish from tutors. Next, the father who had his son destined for the ministry enrolled him in the Atheneum Gedanense (an academic gymnasium), where he focused on Lutheran Theology, Greek and oriental languages. In 1642, according to his parents’ wishes, Schmiedt got a place at the University of Königsberg to study theology. However, within a few months, he changed his academic focus to medicine because it had attracted him the most. In January 1646, benefiting from a vibrant intellectual atmosphere at the Medical Faculty, he held a so-called practice disputation (*disputatio exercitii gratia*), moving shortly thereafter to the Dutch Republic to continue his medical training in Leiden under the guidance of Johannes Walaeus and Otto van Huern.

The next stops on Schmiedt’s educational journey were the Southern Netherlands and France. In France, he visited Paris first, and only later, brimming with enthusiasm, he matriculated at the University of Montpellier, which was known for its numerous facilities for clinical, pharmacological and anatomical teaching. As a student demonstrating extraordinary motivation and talent, he was soon invited to live under one roof with Lazarus Rivière, the personal physician of the King of France, the distinguished proponent of iatrochemistry and leading professor at the Medical Faculty. In 1648, as his supervisee, Schmiedt received a bachelor’s degree, and, in February 1649, he earned his doctorate [1]. Soon thereafter he headed for Italy to take individual instruction from the most eminent anatomists of the day. For example, he stayed for a few months in Padua to train under Johannes Vesling.

After his eight-year *peregrinatio medica* the 27-year-old graduate went back to his homeland and set up a private medical practice, enjoying successes and also enduring mistakes. In July 1661, the municipality appointed him *physicus ordinarius* (city physician). Together with Johann Ernst Scheffler, he began compiling the Danzig municipal pharmacopoeia at the behest of the Council. Just a year later, its outline appeared in print [2]. The pharmacopoeia was completed in 1665, although it was not to be published. In turn, in the summer of 1668, the local pharmaceutical taxa (price list) was printed [3]. All these documents were drawn up under Schmiedt’s supervision who also tried to establish a learned society to promote medicine and natural history in Danzig, but this was to no avail.

Schmiedt married twice, and with his second wife, he had five children. In 1686, his only son, who was to carry on the family medical tradition, died prematurely in Helmstedt and was buried there. Having lost a male descendant, sadly the doctor broke down, his health deteriorated, and he passed away on March 3rd, 1690.

Undoubtedly, Schmiedt was a prolific and capable physician with a long-established reputation. He published numerous scientific papers, documenting his daily medical practice and the experiments he carried out at local hospitals. He and his cordial friend an astronomer Johannes Hevelius sent these original case study reports to the “Philosophical Transactions of the Royal Society” and “Miscellanea Curiosa” of the Academy of the Curious as to Nature (currently the Leopoldina), whereas the “Journal des Sçavans”, the earliest academic journal in Europe, published French translations of Schmiedt’s case studies that had originally been printed in English.

In the 1670 s, Schmiedt described a few cases of hypoesthesia and dysphagia in patients with nervous and mental disorders. Moreover, as a medical examiner, he always interpreted odd human behaviours as exhibitions of organic diseases like epilepsy, ‘melancholy’ and ‘hysteria’ rather than as a display of demonic possession [4, 5]. He also made several noteworthy contributions to the semiology of aphasia. In 1676, he provided the first definite description of a paraphasic expressive speech disorder in a stroke patient with right-sided paralysis. The cerebrovascular patient could not recognize letters and read words, yet he could write them down when dictated. It was the earliest description of pure alexia. Furthermore, Schmiedt proved that the course of alexic deficit might vary from patient to patient [6]. In another report, dated 1672, he extensively described conversion mutism in an adult [7].

Schmiedt also pioneered intravenous (IV) injections and infusions in the treatment of epileptic patients and patients with neurosyphilis. In December 1665, he carried out his first experiment on a soldier with tertiary syphilis and a woman suffering from ‘falling sickness,’ after being admitted to a local hospital. In 1666, he described the successful IV therapy of two women who developed epilepsy in early childhood. In January 1668, in turn, he applied IV injections in the therapy of patients with post-stroke seizures and Polish plait. Consequently, the ‘apoplectic’ patient had no relapses of convulsive episodes. Also, in a patient suffering from *plica polonica*, the previously reported neurologic manifestations of the disease receded and did not recur [8, 9]. Schmiedt carefully described his medical investigation, discussing its results in unprinted private letters to scholars in Germany, England and France.

From the standpoint of neurology, the crowning achievement of Schmiedt’s medical career was the IV administration of antiepileptic drugs. In a letter sent in November 1669 from Danzig to London, Hevelius wrote to Henry Oldenburg, the secretary of the Royal Society: ‘I have no doubt but that it will be a great boon to the whole human race if this therapy is adopted pretty commonly in different regions of the globe’ [10]. And indeed, in modern neurology, IV and intramuscular formulations of anti-seizure drugs are crucial in the clinical treatment of seizure emergencies as well as in replacement therapy when oral administration is not possible or effective.
